# Identification of mitochondrial-related genes as potential biomarkers for the subtyping and prediction of Alzheimer’s disease

**DOI:** 10.3389/fnmol.2023.1205541

**Published:** 2023-07-04

**Authors:** Wenhao Ma, Yuelin Su, Peng Zhang, Guoqing Wan, Xiaoqin Cheng, Changlian Lu, Xuefeng Gu

**Affiliations:** ^1^School of Pharmacy, Shanghai University of Medicine and Health Sciences, Shanghai, China; ^2^Shanghai Key Laboratory of Molecular Imaging, Shanghai University of Medicine and Health Sciences, Shanghai, China; ^3^School of Health Science and Engineering, University of Shanghai for Science and Technology, Shanghai, China; ^4^Department of Ultrasound Medicine, Huashan Hospital Affiliated to Fudan University, Shanghai, China; ^5^Department of Neurology, Zhongshan Hospital, Fudan University, Shanghai, China

**Keywords:** Alzheimer’s disease, mitochondrial autophagy, mitochondrial-related genes, biomarkers, prediction model, subtyping

## Abstract

**Introduction:**

Alzheimer’s disease (AD) is a progressive and debilitating neurodegenerative disorder prevalent among older adults. Although AD symptoms can be managed through certain treatments, advancing the understanding of underlying disease mechanisms and developing effective therapies is critical.

**Methods:**

In this study, we systematically analyzed transcriptome data from temporal lobes of healthy individuals and patients with AD to investigate the relationship between AD and mitochondrial autophagy. Machine learning algorithms were used to identify six genes—*FUNDC1*, *MAP1LC3A*, *CSNK2A1*, *VDAC1*, *CSNK2B*, and *ATG5*—for the construction of an AD prediction model. Furthermore, AD was categorized into three subtypes through consensus clustering analysis.

**Results:**

The identified genes are closely linked to the onset and progression of AD and can serve as reliable biomarkers. The differences in gene expression, clinical features, immune infiltration, and pathway enrichment were examined among the three AD subtypes. Potential drugs for the treatment of each subtype were also identified.

**Discussion:**

The findings observed in the present study can help to deepen the understanding of the underlying disease mechanisms of AD and enable the development of precision medicine and personalized treatment approaches.

## 1. Introduction

Alzheimer’s disease (AD) is a debilitating neurodegenerative disorder characterized by a progressive decline in memory and cognitive function. The underlying mechanisms of AD require further elucidation in spite of considerable research on the subject, and current therapeutic options are limited (Lane et al., 2018). Recently, alterations in cellular energy metabolism, including defects in mitochondrial function, were reported to influence AD pathogenesis (Moreira et al., 2010; Wang et al., 2020).

Mitochondrial autophagy, also known as mitophagy, is a crucial cellular process involved in maintaining cellular homeostasis and energy metabolism. Mitophagy is a process of selective degradation through which damaged or surplus mitochondria are targeted for removal, thereby promoting mitochondrial turnover and preventing the accumulation of damaged mitochondria (Kim et al., 2007). Reportedly, AD is characterized by the accumulation of damaged neuronal mitochondria and increased oxidative stress caused as a result of the impairment of mitophagy (Smith et al., 2000; Manczak et al., 2006; Kerr et al., 2017; De Gaetano et al., 2021). In addition, mitophagy dysfunction inhibits ATP production and activates AMPK. Excessive activation of AMPK further reduces ATP production and induces tau protein phosphorylation, which is crucial for Aβ synaptic toxicity. Additionally, impaired mitophagy has a negative impact on microglia. Microglia play a key role in clearing neurotoxic protein components; however, when mitophagy is impaired, microglia cannot effectively phagocytize and remove Aβ plaques, resulting in the accumulation of Aβ (Leuner et al., 2012; Fang et al., 2019; Song et al., 2021); these are typically considered characteristic features of AD. Furthermore, mitophagy is known to be affected by AD-associated genetic mutations, including those in the presenilin 1 gene (Martin-Maestro et al., 2017), highlighting a direct connection between AD and mitophagy. Promoting mitophagy can improve cognitive function in AD animal models, and drugs targeting the mitophagy pathway may have therapeutic potential for the treatment of AD (Cen et al., 2020, 2021; Chen et al., 2021; Liang et al., 2021; Wang et al., 2021).

These findings highlight the fact that the relationship between AD and mitophagy has progressively garnered attention as an important field of research with immense potential to improve the current understanding of the underlying mechanisms of AD and to develop novel therapeutic strategies.

The purpose of the present study was to gain a deeper understanding of the relationship between AD and mitophagy through a systematic analysis of transcriptomic data from the middle temporal gyrus (MTG) in healthy individuals and patients with AD. Machine learning algorithms were utilized to construct prediction models for AD based on 27 mitophagy-related genes (MRGs). These models were evaluated in terms of their performance through multiple validation techniques, including receiver operating characteristic (ROC) curves, calibration curves, nomograms, decision curve analyses (DCA), and external datasets. Additionally, three subtypes of AD were developed through consistent cluster analysis, the biological functions, and pathways specific to each subtype were compared, and interpatient differences were analyzed in terms of age and sex in each subtype. Weighted gene co-expression network analysis (WGCNA) was used to obtain hubgenes, and the Connectivity Map (CMap) database was utilized to identify potential small molecule drugs that may target each subtype.

## 2. Materials

### 2.1. Data acquisition and processing

The NCBI Gene Expression Omnibus (GEO) public database^1^ was used to search for gene expression data, and these data were obtained using the “GEOquery” R package. The data comprised two datasets, GSE109887 (GPL10904 platform) and GSE132903 (GPL10558 platform), which were merged to form a total of 130 and 143 samples from healthy individuals and patients with AD, respectively. The samples were collected from the MTG, a site of early AD pathology (Ray and Zhang, 2010; Chen et al., 2022). Merged data were processed to eliminate batch effects from different platforms and to normalize the data using the “sva” package. A principal component analysis (PCA) was subsequently performed to assess data combinations. The performance of the prediction model was validated using the GSE5281 dataset (GPL570 platform), which contained 74 and 87 samples from healthy individuals and patients with AD, respectively, and the samples were obtained from several brain regions including the entorhinal cortex, hippocampus, medial temporal gyrus, posterior cingulate, superior frontal gyrus, and primary visual cortex.

The mitophagy-associated gene set “REACTOME_MITOPHAGY.v7.5.1.gmt” was procured from the Reactome database. Meanwhile, the gene set “BIOCARTA_INFLAM_PATHWAY.v7.5.1.gmt,” which is associated with inflammation factors, was acquired from the Biocarta database.

### 2.2. Gene set variation analysis

The gene set variation analysis (GSVA) was performed using the “GSVA” R package to investigate the differences in the expression of MRGs between patients with AD and healthy individuals and between subtypes of AD. The results were visualized using the “ggpubr” package to clearly demonstrate the variations in gene expression.

### 2.3. Identification of differentially expressed MRGs

Differential gene expression between samples was determined using the “limma” package, with a stringent criterion of *p* < 0.05. The MRGs with differential expression in patients with AD were determined by taking into consideration the intersection of the differentially expressed genes (DEGs) and MRGs. The DEGs were visualized as a volcano plot and heatmap generated using the “ggplot2” and “pheatmap” packages, respectively.

### 2.4. Construction and validation of prediction models

The 27 MRGs were screened using the random forest method, and the characteristic genes were selected according to the minimum cross-validation error achieved from the ntree = 1,000 iteration. The importance score of the selected characteristic genes was subsequently evaluated. The selection process was made more specific through the application of a stepwise regression algorithm (Gu et al., 2022) to the top 10 genes. The prediction models were established through multifactor logistic regression and the graphical representation of the model was depicted using the forest plot obtained from the R package “forestplotter.” Screening and modeling processes were implemented using the “randomForest” and “caret” R packages, respectively. ROC curves were constructed using the R package “pROC,” and the area under the ROC curve (AUC) of the prediction model was determined. Furthermore, a nomogram model was established using the “rms” R package. The predictive power of each characteristic gene was quantified by assigning a predictive power score, and the total score represented the sum of the predictive power scores for all characteristics in the prediction model. The predictive power of the line graph model was evaluated by using calibration curves and DCA (Lai et al., 2021). The validity of these results was verified with an external dataset.

### 2.5. Analysis of immune infiltration and expression levels of inflammatory factor genes

The relative proportion of infiltrated immune cells was quantified using the MCPcounter algorithm. MCPcounter is a transcriptomic-based quantitative method that measures the absolute abundance of eight immune cell populations (T cells, CD8 T cells, Cytotoxic lymphocytes, B lineage, NK cells, Monocytic lineage, Myeloid dendritic cells, and Neutrophils) and two stromal cell populations (Endothelial cells, Fibroblasts) in heterogeneous tissues. The method relies on cell type-specific gene expression values to derive an abundance score for each individual cell type and sample, allowing for direct comparison of cell type abundance across different experimental conditions. Additionally, the expression levels of inflammatory factor genes were analyzed in the samples from healthy individuals and patients with AD, as well as in subtypes, to detect differential expression. The correlation between MRGs and infiltrated immune cells and inflammatory factors was explored using Spearman’s correlation coefficient.

### 2.6. Consensus clustering analysis

Unsupervised clustering analysis was performed on the 143 samples from patients with AD using the “ConsensusClusterPlus” R package according to the expression profiles of 27 MRGs. The K-means algorithm was utilized, with a maximum subtype number set at 10 (*k* = 10), a sampling ratio of 0.8, and 100 re-sampling times. The optimal number of clusters was determined through the evaluation of the cumulative distribution function (CDF) curve, tracking plots, consensus matrix, delta area, and consistent cluster score (>0.9) based on the results of consensus clustering.

### 2.7. Analysis of enrichment

The gene ontology (GO) and Kyoto Encyclopedia of Genes and Genomes (KEGG) pathway analysis of DEGs among subtypes was performed using the “clusterProfiler” R software package.

### 2.8. Weighted gene co-expression network analysis

Weighted gene co-expression network analysis was performed using the “WGCNA” R package for the identification of the hub genes for each subtype. The genes in the top 5,000 of median absolute deviation (MAD) were initially selected, and a similarity matrix was constructed by computing the correlation coefficients between each pair of genes. The soft threshold of 12 was subsequently used for the conversion of a similarity matrix into an adjacency matrix, and further into a topological overlap matrix (TOM) to evaluate the average network connectivity of each gene. The “blockwiseModules” functions (minModuleSize = 30, mergeCutHeight = 0.25) were used to categorize genes with similar expression profiles into modules, and each module was identifiable with a unique color. The module signature genes (ME) represented the gene expression profile of each module and facilitated the authors in accurately assessing the relationship with the phenotype. Module membership (MM) represents the relevance of the gene to the module through the correlation coefficient with the gene expression values and ME. ME and MM were used to identify crucial subtype-specific modules.

### 2.9. Identification of potential small molecule drugs

Potential subtype-specific small molecule drugs for the treatment of AD were identified by obtaining the intersection of core genes and DEGs for each subtype. The top 150 upregulated and downregulated genes with the highest fold change were subsequently inputted into the Connectivity Map (CMAP) database.^2^ The purpose of this search was the identification of drugs with potentially beneficial effects on the treatment of AD according to subtype. The drug scores ranged from −100 to 100; a lower score indicated a greater potential for the drug in terms of its applications in the treatment of the corresponding AD subtype.

## 3. Results

### 3.1. Dysregulation of mitochondrial autophagy and activation of the immune system in AD

**Figure 1:**
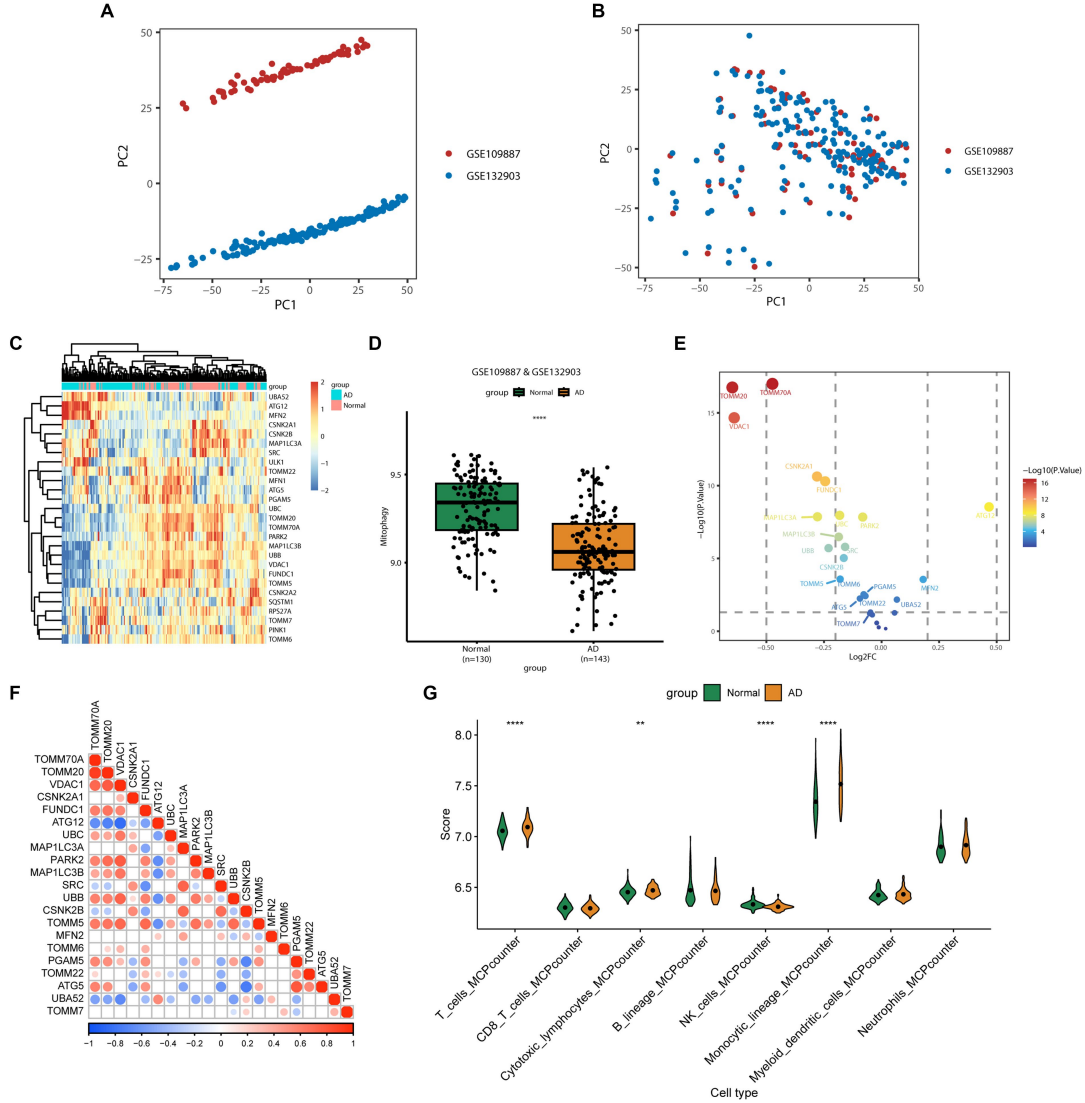
Differentially expressed mitophagy-related genes (MRGs) in Alzheimer’s disease (AD). **(A)** Principal component analysis (PCA) of GSE109887 and GSE132903 datasets before batch effect removal. **(B)** PCA of GSE109887 and GSE132903 datasets after batch effect removal. **(C)** The expression patterns of 27 MRGs are presented in the heatmap. Each row represents a specific MRG and each column represents a sample. The color gradient ranging from blue to red indicates low to high expression levels, respectively. **(D)** The box plot displays the GSVA scores of the MRG gene set across samples to observe the overall expression differences of MRG between healthy individuals and patients with AD. *p* values were estimated by Student’s *t*-test. ^****^p < 0.0001. **(E)** The volcano map illustrates 21 differentially expressed MRGs in AD, where a negative log2FC indicates downregulation and a positive log2FC indicates upregulation of the gene expression. **(F)** Correlation analysis between the 21 differentially expressed genes. Red and blue represent positive and negative correlations, respectively. The depth of color represents different correlation coefficients. **(G)** The violin diagram shows the difference in infiltrated immune cells between patients with AD and healthy subjects. The horizontal axis represents the immune cell types, while the vertical axis denotes the scores calculated by the mcpcounter algorithm. *p* values were estimated by Student’s *t*-test. ^**^p < 0.01, ^****^p < 0.0001.

The expression profiles of 27 MRGs were compared between patients with AD and normal controls using the GSE109887 and GSE132903 datasets (Figures 1A,B). Typically, MRGs appeared to be downregulated in patients with AD (Figures 1C,D), and this result was validated by the GSE5281 dataset (Supplementary Figure 1A). Analysis of differential expression revealed 21 DEGs, of which 18 were downregulated (*TOMM20*, *VDAC1*, *TOMM70A*, *CSNK2A1*, *MAP1LC3A*, *FUNDC1*, *UBB*, *MAP1LC3B*, *UBC*, *TOMM5*, *CSNK2B*, *SRC*, *ATG5*, *PARK2*, *TOMM6*, *PGAM5*, *TOMM22*, and *TOMM7*) and three were upregulated (*UBA52*, *MFN2*, and *ATG12*; Figure 1E, Supplementary Figure 1B). Correlation analysis revealed that *TOMM20*, *PARK2*, *SRC*, and *CSNK2A1* were positively correlated with multiple genes from the 21 differentially expressed mitophagy-related genes, while *UBA52* and *ATG12* were negatively correlated with multiple genes from the same gene set (Figure 1F). Inflammation factor expression analysis showed that CD4, CSF1, CSF3, IFNA1, and IL1A were downregulated in AD, whereas HLA-DRB3, HLA-DRB4, IL10, IL15, IL5, IL6, PDGFA, and TGFB3 were upregulated (Supplementary Figure 1C). Analysis of immune cell infiltration using the MCPcounter algorithm showed that the proportions of T cells, cytotoxic lymphocytes, and monocytes increased in patients with AD, whereas that of NK cells decreased (Figure 1G).

### 3.2. Construction and evaluation of predictive models

**Figure 2:**
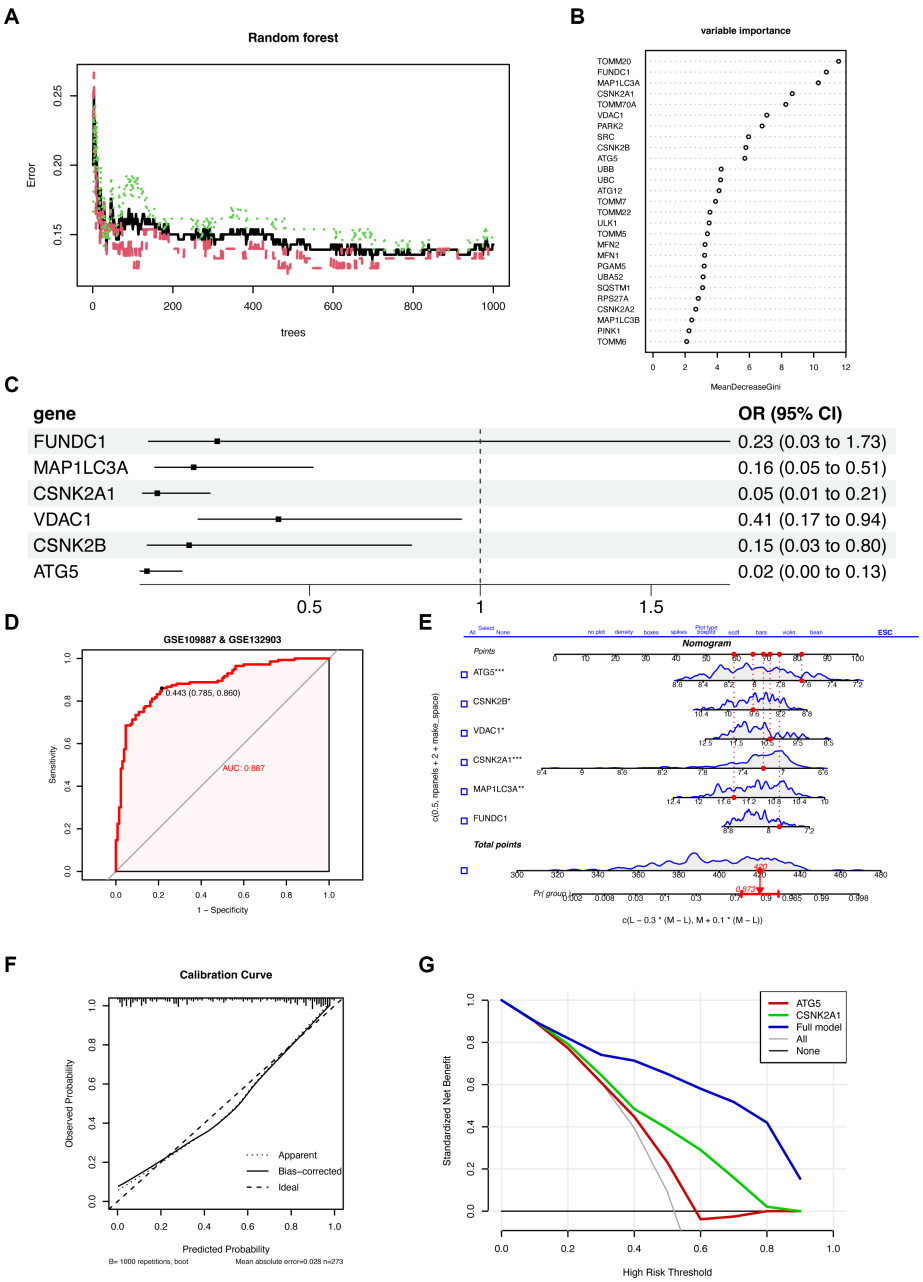
The establishment and evaluation of the predictive model. **(A)** Cumulative residual distribution of random forest learning model. The horizontal axis represents the number of trees (decision tree classifiers), while the vertical axis represents the corresponding prediction error under that number of trees. The plot demonstrates the variation of prediction error of the Random Forest learning model under different quantities of trees. **(B)** Ranking plot depicting the variable importance in random forest. The MeanDecreaseGini index is used to measure the importance of feature genes in the model, where a higher value indicates a higher level of importance of that variable in the model. **(C)** Forest plot demonstrating the six variables selected through stepwise logistic regression screening. **(D)** Receiver operating characteristic curve evaluating the diagnostic performance of feature genes. The horizontal axis represents the false positive rate, while the vertical axis represents the true positive rate. A higher AUC indicates a higher accuracy of the model in diagnosing the disease. **(E)** Nomogram for predicting the risk of AD based on feature genes. The feature weight of six important feature genes is used as input variables, where the “Points” refer to the corresponding scores of each input variable, and the “Total points” denote the total score obtained by adding up the scores of all input variables. “Pr (group)” represents the risk score corresponding to the “Total points,” which indicates the likelihood of developing AD. **(F)** Calibration curve illustrating the calibration performance of a predictive model. The horizontal axis represents the predicted value, while the vertical axis represents the actual observed value. The closer the bias-corrected curve is to the Ideal dashed line, the higher the calibration performance of the model. **(G)** DCA estimates the clinical benefit of the nomogram. The plot shows a comparison of the net clinical benefits for different prediction models at different decision thresholds. The blue model, which is constructed using six feature genes, performs the best, with the highest net clinical benefit relative to the optimal treatment reference line.

Multiple machine learning algorithms (Random Forest, Stepwise Regression, and Multivariate Logistic Regression) were used to screen the 27 MRGs genes and construct a predictive model for AD. First, the 27 genes were inputted into the Random Forest classifier, and the correlation between the model error and the number of random forest trees revealed that the error rate was the most stable when using 698 trees (Figure 2A). The top 10 genes, determined on the basis of importance ranking, were selected for further analysis using Stepwise Regression and Multivariate Logistic Regression (Figure 2B). The results of these algorithms showed that *FUNDC1*, *MAP1LC3A*, *CSNK2A1*, *VDAC1*, *CSNK2B*, and *ATG5* were the six remaining genes (Figure 2C). Furthermore, their odds ratios were < 1, which suggested that they could be considered protective factors for AD. The predictive performance of these six genes was verified through ROC analysis (AUC = 0.887; Figure 2D), and a validation set ROC analysis (AUC = 0.843; Supplementary Figure 2A), which highlighted the model’s excellent performance in terms of predicting AD. A scoring model was consequently established to evaluate the probability of AD based on the expression of the abovementioned six genes (Figure 2E). Calibration and DCA curves were constructed and analyzed using the validation set, and the Apparent line and Bias-Corrected line in the calibration curve appeared to have a good fit with the Ideal line (Figure 2F, Supplementary Figure 2B). The DCA curve showed that the model had higher net profit when all the selected feature genes were used (Figure 2G, Supplementary Figure 2C), indicating that the model is reliable for predicting AD. Correlation analyses were subsequently performed between these six feature genes and the coding genes of amyloid precursor protein (APP) and tau protein (MAPT); these genes are closely associated with AD development and progression. The results showed that the expression of these six genes was positively correlated with both *APP* and *MAPT* (Supplementary Figures 3A,B), suggesting that these genes have exceptional predictive value for AD.

### 3.3. Identification of mitochondrial autophagy subtypes in AD

**Figure 3:**
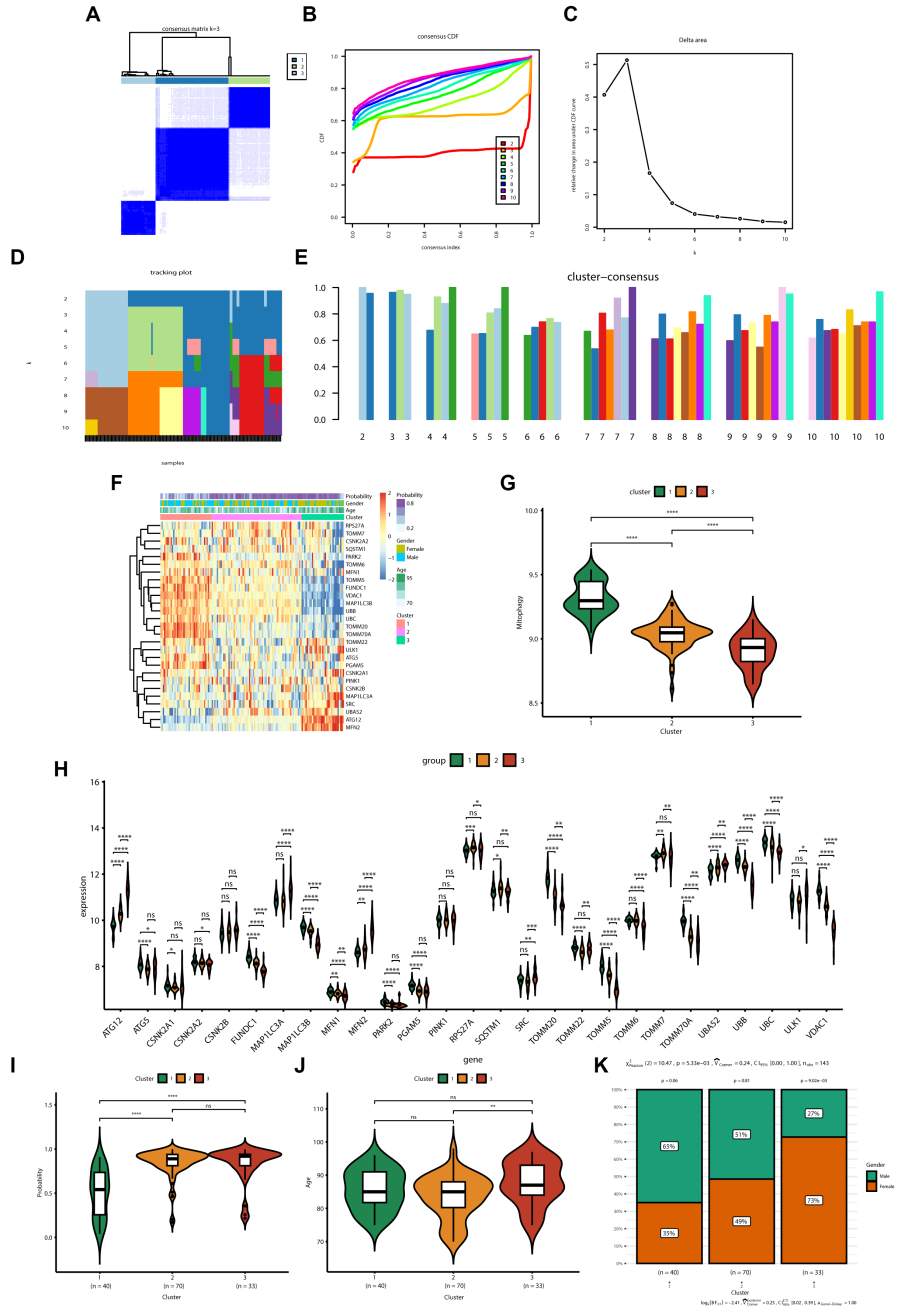
Identification of the subtypes of Alzheimer’s disease and differences between subtypes. **(A)** Consensus clustering matrix showing the clustering agreement between samples when *k* (number of clusters) = 3. **(B)** Representative cumulative distribution function (CDF) curve showing the clustering results for *k* (number of clusters) ranging from 2 to 10. **(C)** Relative changes in CDF delta area curves, which measure the stability of clustering across different values of *k* (number of clusters). **(D)** Tracking plot illustrating the classification of samples into different subtypes based on the clustering results obtained using different values of *k* (number of clusters). Each sample is assigned a different color based on its membership in different clusters. **(E)** Consensus scores for each subtype when k (number of clusters) ranges from 2 to 10. The *x*-axis represents the number of clusters, while the *y*-axis represents the consensus score for each cluster. A higher consensus score indicates a more robust clustering result. **(F)** Heatmap depicts the expression of 27 mitochondrial autophagy-related genes in three subtypes, with the addition of clinical information. The “Probability” shown in the color scale represents the likelihood that the patient be diagnosed with AD based on a diagnostic model constructed using six genes. **(G)** The boxplot displays the overall expression of MRGs among the three subtypes (cluster 1, cluster 2, and cluster 3). *p* values were estimated by Student’s *t*-test. ^****^p < 0.0001. **(H)** The expression levels of 27 MRGs were compared between three clusters (cluster 1: *n* = 40, cluster 2: *n* = 70, cluster 3: *n* = 33) using Student’s *t*-test. ^*^p < 0.05, ^**^p < 0.01, ^***^p < 0.001, ^****^p < 0.0001; ns, no significance. **(I)** Comparison of the diagnostic performance of the prediction models for the three subtypes (cluster 1: *n* = 40, cluster 2: *n* = 70, cluster 3: *n* = 33). *p* values were estimated by Student’s *t*-test. ^****^p < 0.0001; ns, no significance. **(J)** Comparison of the age of patients with AD in the three subtypes (cluster 1: *n* = 40, cluster 2: *n* = 70, cluster 3: *n* = 33). *p* values were estimated by Student’s *t*-test. ^**^p < 0.01; ns, no significance. **(K)** Gender distribution of patients with AD in three subtypes.

The consensus clustering method was applied to samples from patients with AD based on the expression profiles of 27 MRGs. The best clustering number was *K* = 3 (Figure 3A), based on a comprehensive evaluation of the CDF curve (Figure 3B), delta area (Figure 3C), tracking plot (Figure 3D), and consistent cluster score (Figure 3E). Differences in the expression of mitochondrial autophagy genes, clinical features (age and sex), and diagnostic model efficacy among the three subtypes were displayed using a heatmap (Figure 3F). The GSVA indicated that the overall expression of MRGs for the clusters was as follows in descending order of magnitude: clusters 1, 2, and 3 (Figure 3G).

The expression of specific genes—including *PARK2*, *ATG5*, *FUNDC1*, *TOMM20*, and *VDAC1*—was significantly lower in clusters 2 and 3 than in cluster 1 (Figure 3H). Furthermore, the AD prediction model constructed in the present study showed a higher predictive ability for clusters 2 and 3 than that for cluster 1 (Figure 3I). Moreover, patients with AD in cluster 3 were significantly older than those in cluster 2 (Figure 3J). In terms of sex, the number of female patients progressively increased from clusters 1 to 3; the number of female patients in cluster 3 was significantly higher than the number of male patients (Figure 3K).

### 3.4. Differences in immune infiltration of the subtypes of mitochondrial autophagy

**Figure 4:**
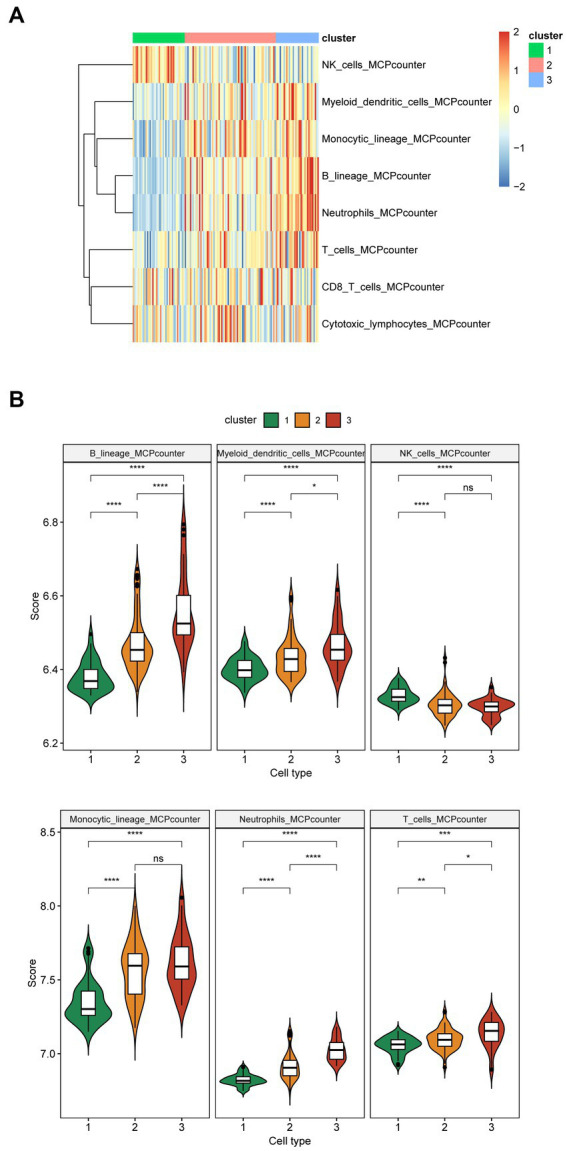
Immune infiltration of the three subtypes of Alzheimer’s disease. **(A)** The heatmap shows overall immune infiltration in the three subtypes. Blue represents relatively low levels of immune cell infiltration, while red represents relatively high levels of infiltration. **(B)** The violin plot shows immune infiltration in the three subtypes (cluster 1: *n* = 40, cluster 2: *n* = 70, cluster 3: *n* = 33). The *x*-axis represents different subtypes, while the *y*-axis represents the scores calculated by the MCPcounter algorithm. Immune cells with no difference in infiltration levels across the three cluster are not displayed. *p* values were estimated by Student’s *t*-test. ^*^p < 0.05, ^**^p < 0.01, ^***^p < 0.001, ^****^p < 0.0001; ns, no significance.

The MCPcounter algorithm was used to analyze immune infiltration across three subtypes. The heatmap displayed the overall differences in immune infiltration among three subtypes (Figure 4A). Specifically, the infiltration levels of B lineage, myeloid dendritic cells, neutrophils, and T cells gradually increased from cluster 1 to 3. Additionally, the infiltration level of monocytic lineage in clusters 2 and 3 was significantly higher than those in cluster 1. Conversely, the infiltration levels of NK cells in clusters 2 and 3 were significantly lower than those in cluster 1 (Figure 4B). In terms of inflammatory factors, cluster 1 exhibited significantly lower expression levels of CD4, HLA-DRB3, IL10, PDGFA, and TGFB3 in contrast to clusters 2 and 3; however, IFNA1 and IL1A were markedly upregulated in cluster 1. Furthermore, Cluster 3 displayed the lowest expression levels of HLA-DRA, HLA-DRB4, L11, IL1A, IL6, and TGFB2, whereas L10, IL5, and PDGFA were significantly upregulated in cluster 3 (Supplementary Figure 4A).

### 3.5. GO and KEGG enrichment analysis of three subtypes

**Figure 5:**
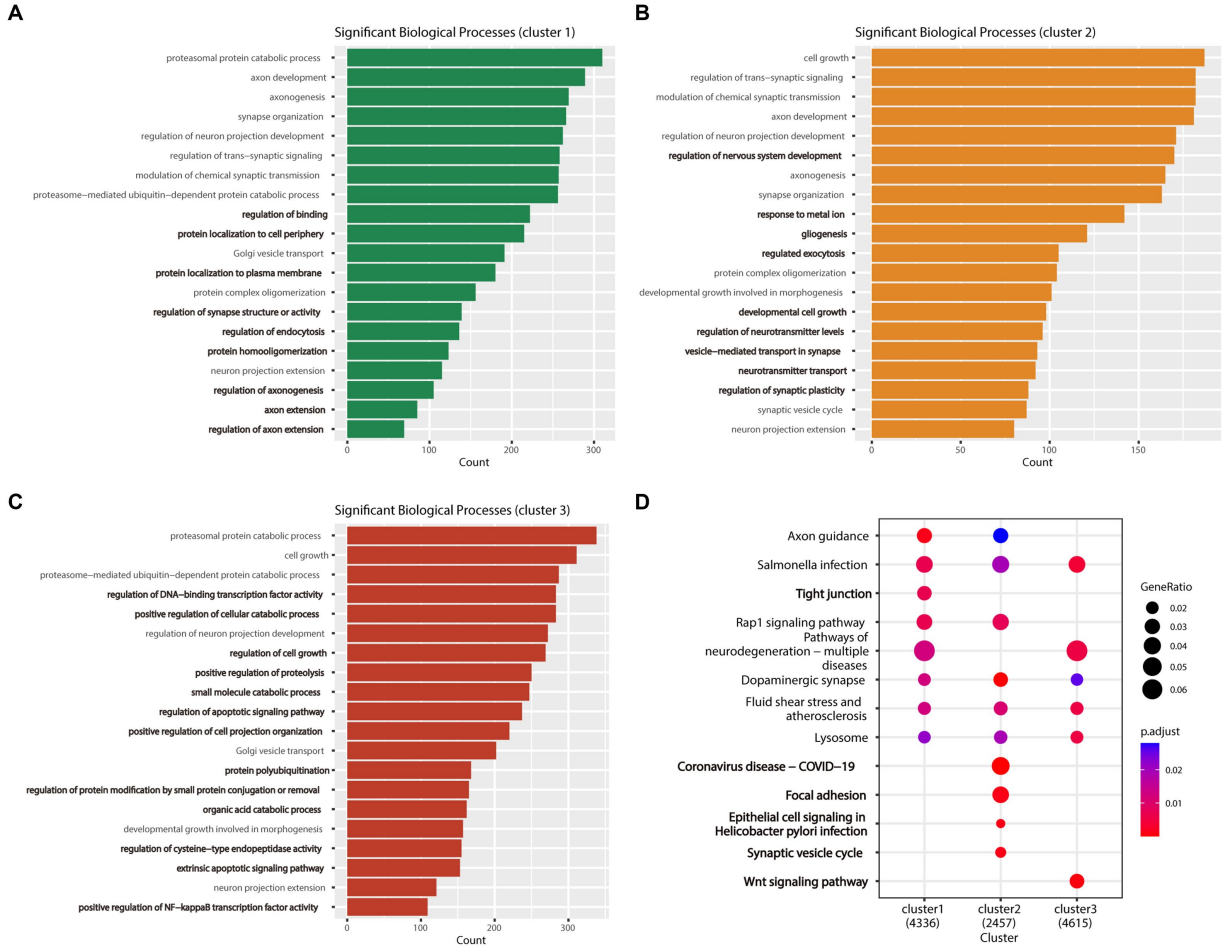
Enrichment analysis results of the three AD subtypes. **(A)** Bar plot of the biological process GO analysis results for Cluster 1, with the biological processes unique to this cluster highlighted in bold. **(B)** Bar plot of the biological process GO analysis results for Cluster 2, with the biological processes unique to this cluster highlighted in bold. **(C)** Bar plot of the biological process GO analysis results for Cluster 3, with the biological processes unique to this cluster highlighted in bold. **(D)** The bubble plot shows the KEGG pathway analysis results for the three subtypes (clusters 1, 2, and 3) combined, with pathways unique to each subtype highlighted in bold.

Gene ontology and KEGG enrichment analyses were performed on DEGs of one subtype relative to the other two subtypes. The findings of GO enrichment analysis demonstrated that the subtypes examined herein exhibited distinct functional characteristics in terms of cellular growth, synaptic organization, and neuronal projection. Although all three subtypes shared certain similarities in the regulating processes related to neuronal projection extension and chemosynaptic transmission, the subtypes were different in terms of their specific biological processes. Cluster 1 was associated with regulation of binding, regulation of synapse structure or activity, and regulation of axonogenesis (Figure 5A), whereas cluster 2 was associated with regulation of nervous system development, developmental cell growth, and vesicle−mediated transport in synapse (Figure 5B). Cluster 3 was involved in positive regulation of cellular catabolic process, regulation of cell growth, and regulation of apoptotic signaling pathway (Figure 5C). Although clusters 1 and 2 shared certain functional characteristics, including protein complex oligomerization and protein localization to the plasma membrane, cluster 3 was predominantly different with regard to its involvement in the organic acid catabolic process and regulation of DNA-binding transcription factor activity.

Kyoto Encyclopedia of Genes and Genomes pathway enrichment analysis of the three subtypes enabled the identification of commonly enriched pathways across all three subtypes, including the dopaminergic synapse, lysosome, and salmonella infection pathways. Notably, each subtype exhibited its unique pathways. Cluster 2 was primarily enriched in pathways related to coronavirus disease infectious disease (COVID-19), epithelial cell signaling in *Helicobacter pylori* infections, focal adhesion, and synaptic vesicle cycle. In contrast, clusters 1 and 3 were associated with the tight junction and the wnt signaling pathways. Furthermore, we found that the rap1 signaling pathway was commonly enriched in clusters 1 and 2, whereas the pathways of neurodegeneration—multiple diseases were commonly enriched in clusters 1 and 3 (Figure 5D).

### 3.6. Screening of hub genes and prediction of small molecule drugs

**Figure 6:**
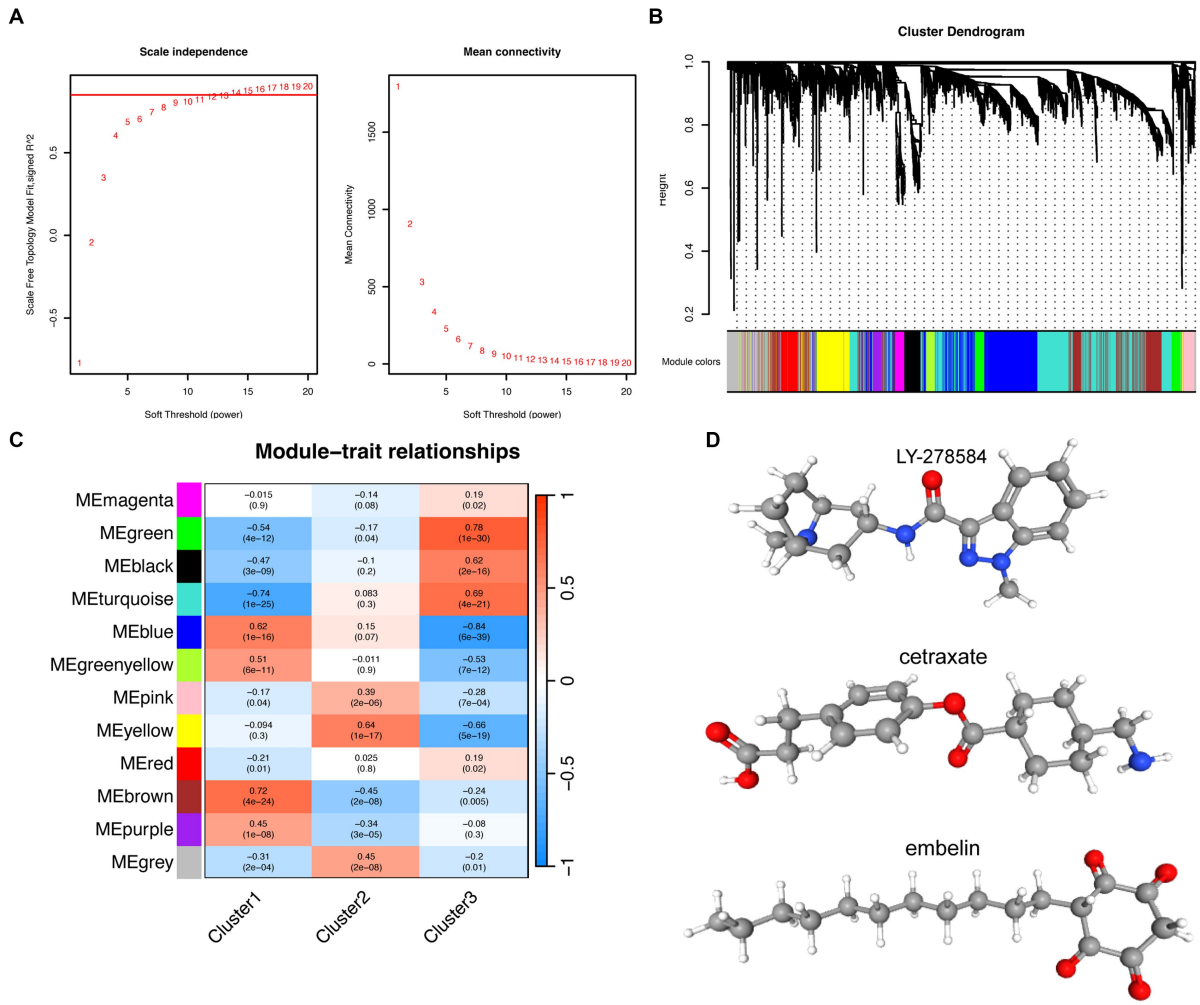
Prediction of small molecule drugs for the three subtypes of Alzheimer’s disease. **(A)** The left panel shows the indices of the scale-free topological model fit for different soft threshold powers, while the right panel displays the corresponding mean connectivity values for each soft threshold power. **(B)** Cluster dendrogram of genes. The leaf nodes (i.e., the bottom-most nodes) of the cluster dendrogram represent individual genes or small clusters, while the top of the tree represents the overall expression pattern of all genes in the gene set. **(C)** Correlations between different modules and clusters. Each row represents a different gene co-expression module, and each column represents a different cluster. The values in the boxes represent the correlation coefficients, with red and blue distinguishing positive and negative correlations, respectively. The values in parentheses represent the significance value of *p*. **(D)** Molecular structures of predicted small molecule drugs: LY-278584, cetraxate, and embelin.

Weighted gene co-expression network analysis was performed to identify key gene modules associated with each subtype of AD. By combining the *R*^2^ values and the Mean Connectivity values, a soft threshold of 12 was selected to construct a scale-free network (Figure 6A), and 12 modules were identified using hierarchical clustering. Each of these modules was represented through different colors, while the gray module represented unassigned genes (Figure 6B). Based on the correlation analysis between each module (excluding the gray module) and clinical features (clusters 1, 2, and 3), we selected the “brown” module as the core module for cluster 1, the “yellow” module for cluster 2, and the “green” module for cluster 3 (Figure 6C). In order to identify hub genes in the core modules associated with each subtype, we used the KME (module eigengene-based connectivity measure) value as a screening criteria. KME measures the inter-gene correlation within a module, and high KME values indicate that a gene is highly connected with other genes in the module and may act as a hub gene. By intersecting the hub genes identified by KME with the DEGs in each subtype, we determined the upregulated and downregulated genes that were most closely associated with each subtype. Because of the input limit of 150 genes for cMap, DEGs exceeding 150 were sorted using fold change, and only the top 150 genes were selected. By combining the findings from cMap analysis and relevant literature, we determined that the molecule with the lowest score was the most promising predicted drug for each subtype. Therefore, LY-278584, cetraxate, and embelin were considered predicted drugs for subtypes 1, 2, and 3, respectively (Figure 6D; Table 1).

**Table 1:** Predicted small molecule drugs of each subtype.

Cluster	Rank	Score	Name	MOA
1	8,559	−99.58	LY-278584	Serotonin receptor antagonist
2	8,552	−96.64	Cetraxate	Mucus protecting agent
3	8,552	−99.19	Embelin	HCV inhibitor, XIAP inhibitor

## 4. Discussion

Alzheimer’s disease, a progressive and debilitating neurodegenerative condition, represents the most common cause of dementia among older adults (Hou et al., 2017). It is a complex disorder and involves multiple etiological factors including genetic and environmental influences (Armstrong, 2013); additionally, the underlying mechanisms of AD remain unclear. Mitophagy in neurons is believed to critically influence AD pathogenesis (Chu, 2019); therefore, the potential of targeting MRGs for the treatment of AD should be explored owing to its significant importance in clinical settings. To this end, MRGs were used to construct a predictive model of AD and refine AD subtypes to further clarify the mechanism underlying the pathogenesis of AD, reveal the heterogeneity of AD, and provide new ideas and methods for understanding and treating AD. Transcriptome data from the MTG of healthy individuals and patients with AD were systematically analyzed to investigate the relationship between AD and mitochondrial autophagy. GSVA revealed that the overall expression of MRGs was downregulated in patients with AD. Specifically, 18 MRGs, including those involved in the TOM complex (*TOMM20*, *TOMM70A*, *TOMM5*, *TOMM6*, *TOMM22*, and *TOMM7*), were significantly downregulated, whereas only three genes (*UBA52*, *MFN2*, and *ATG12*) were upregulated. The TOM complex was reported to play a crucial role in mitochondrial protein transport and localization, as well as in the synthesis and maintenance of mitochondrial homeostasis (Wang et al., 2021). Therefore, downregulation of the expression of this complex may result in mitochondrial dysfunction (Chai et al., 2018). In light of these findings, it is evident that changes in the expression levels of genes related to mitochondrial autophagy may play a critical role in AD development and progression.

Developing a predictive model for AD based on MRGs is a promising approach to elucidate the underlying mechanisms of AD and to develop new treatment strategies. Defects in mitochondrial autophagy were reported to critically influence AD pathogenesis. However, to the best of our knowledge, few studies have attempted to systematically investigate the potential utility of MRGs in predicting AD. The purpose of the present study was to address this paucity of information through the use of using machine learning algorithms to identify six genes—*FUNDC1*, *MAP1LC3A*, *CSNK2A1*, *VDAC1*, *CSNK2B*, and *ATG5*—out of 27 MRGs, to construct a prediction model for AD. *FUNDC1* is a mitochondrial outer membrane protein that critically affects the regulation of mitochondrial quality control and metabolism. *FUNDC1* is reported to play an important role in several diseases, such as cancer, cardiovascular disease, and neurological disorders, including AD (Chen et al., 2016). In an *in vitro* model of hippocampal neurons obtained from acquired epilepsy, low *FUNDC1* expression significantly increased neuronal apoptosis (Zhang et al., 2023). The downregulation of *FUNDC1* levels in cases of AD in particular may lead to mitochondrial dysfunction, accelerating the AD progression (Jetto et al., 2022). MAP1LC3A, also known as LC3A, is a protein associated with mitophagy and autophagy. This protein binds to autophagosomes formed during the autophagy process and participates in waste degradation after the fusion of autophagosomes and lysosomes (Cherra et al., 2010; Iriondo et al., 2022). VDAC1—an ion channel on the mitochondrial membrane—is closely related to mitochondrial function and stability (Hiller et al., 2008). VDAC1 can interact with Aβ and phosphorylated tau, which are associated with the deposition and toxicity of Aβ, leading to mitochondrial dysfunction and cell apoptosis. In addition, VDAC1 has a critical role in neuronal development and synapse formation, which implies that it is potentially involved in AD onset and progression (Marin et al., 2007; Cuadrado-Tejedor et al., 2011; Manczak and Reddy, 2012; Reddy, 2013; Smilansky et al., 2015). *CSNK2A1* and *CSNK2B* encode the α and β subunits, respectively, of the protein kinase CK2, which plays a vital role in regulating Aβ deposition and MAPT phosphorylation in AD (Borgo et al., 2021). *ATG5* is an important gene in the autophagy pathway; it participates in the formation and regulation of intracellular membrane structures. During the process of autophagy, ATG5 forms a complex with ATG12 and participates in the formation and degradation of autophagic vesicles (Codogno and Meijer, 2006; Walczak and Martens, 2013). The autophagy pathway was reported to play an important role in AD onset and progression (Li et al., 2010), and ATG5 is thought to be closely associated with the occurrence and development of AD. In light of this information, multiple validation techniques were used in the present study to evaluate the performance of these models. Across different datasets, the AUC values were 0.887 and 0.843, respectively. Our models demonstrated high predictive capability for AD. Furthermore, we found a positive correlation between these six genes and the genes encoding APP and MAPT, suggesting that *FUNDC1*, *MAP1LC3A*, *CSNK2A1*, *VDAC1*, *CSNK2B*, and *ATG5* have the potential to be used as reliable biomarkers to successfully establish AD diagnosis.

Consistent cluster analysis was used to classify AD into three subtypes based on the expression profiles of MRGs, and the differences between these subtypes were analyzed in terms of gene expression, clinical features, immune infiltration, and pathway enrichment. Notably, MRGs exhibited significant differences among the three subtypes. Among the six diagnostic genes, *FUNDC1* and *VDAC1* showed significant differences in terms of their expression in each subtype, both genes showed the highest expression levels in cluster 1, intermediate expression levels in cluster 2, and low expression in cluster 3. Considering that these diagnostic genes are protective factors for AD, a higher expression level is associated with a lower risk of developing AD. Moreover, high levels of *FUNDC1* and *VDAC1* may promote cellular autophagy (Xie et al., 2021; Liu et al., 2022), which could help eliminate the deposition of harmful proteins, including Aβ and MAPT. Furthermore, the predictive model presented in the current study appeared to be significantly better in terms of diagnostic performance for the subtypes with relatively low MRGs expression levels, i.e., clusters 2 and 3, than that for the subtype with higher MRGs expression levels, i.e., cluster 1.

The activation of the immune system is believed to alter central nervous system inflammatory mechanisms and increases amyloid protein load, which may lead to AD onset and progression (Heppner et al., 2015). In the current study, the analysis of differences in immune cells and inflammatory factors between patients with AD and healthy individuals showed that the proportions of T cells, cytotoxic lymphocytes, and monocytic lineage were increased in cases of AD, whereas NK cells were decreased. Regarding inflammatory factors, upregulated expression of HLA-DRB3, HLA-DRB4, IL10, IL15, IL5, IL6, PDGFA, and TGFB3 and downregulated expression of CD4, CSF1, CSF3, IFNA1, and IL1A was observed. Therefore, the immune system of patients with AD is dysregulated and may contribute to the pathological process of AD. Similar to the expression of MRGs, it is imperative to highlight that several immune cells—including B lineage, monocytic lineage, neutrophils, and T cells—exhibited significant intersubtype differences. The infiltration level was highest in cluster 3, followed by cluster 2, and lowest in cluster 1. Furthermore, the proportion of myeloid dendritic cells was significantly lower in cluster 1 than in cluster 2 and cluster 3. The exact role of B cells in AD requires further elucidation. Although B cells can produce immunoglobulins that slow the progression of AD, B cells in the brain may produce pro-inflammatory cytokines that promote AD-associated neuroinflammation and disease progression. Additionally, animal models have shown that B cell activation and infiltration in the brain are associated with AD, and therapeutic depletion of B cells can reverse AD progression (Kim et al., 2021). Neutrophils have been reported to influence AD progression by promoting Aβ pathology and cognitive impairment. The removal or inhibition of neutrophils in AD mouse models can significantly improve cognitive function, reduce Aβ plaque burden and neuronal damage, inhibit neuroinflammation, and restore cerebral blood flow and blood–brain barrier integrity. These results suggest that neutrophils are involved in the promotion of AD and are positively correlated with disease severity (Zenaro et al., 2015; Dong et al., 2018; Stock et al., 2018). The role of T cells in AD remains unclear; however, animal experiments have revealed that cerebral amyloidosis promotes T cell infiltration (Ferretti et al., 2016). These findings are consistent with our research results, which indicated that infiltration levels of these cells are higher in patients with AD with lower levels of mitochondrial autophagy.

According to the results of GO and KEGG enrichment analysis, the three subtypes of AD exhibit differences in specific biological processes and pathways. In terms of biological processes, the three subtypes show distinct functional characteristics in terms of cell growth, synaptic tissue, and neuron projection. KEGG analysis revealed that cluster 2 is enriched in COVID-19, epithelial cell signaling in *H. pylori* infection, focal adhesion, and synaptic vesicle cycle. SARS-CoV-2—the causative agent of COVID-19—has the ability to attack the central nervous system (Desforges et al., 2019) and may accelerate brain aging and promote the development of neurodegenerative diseases (Ciaccio et al., 2021). Although a definitive correlation between SARS-CoV-2 infection and susceptibility to AD does not exist, the risk of AD was reported to be significantly increased in older adults with COVID-19 (Wang et al., 2022). Our results further support the potential link between AD and COVID-19. “Epithelial cell signaling in *H. pylori* infection” denotes the cellular signal transduction pathway activated by *H. pylori* infection, which is associated with chronic gastritis and gastric cancer (Naumann and Crabtree, 2004). Notably, a link between *H. pylori* infection and AD has been reported, with some studies suggesting that *H. pylori* infection may be implicated in the pathophysiology of AD (Doulberis et al., 2018). Accordingly, the enrichment of signaling pathways related to *H. pylori*-infected epithelial cells in Cluster 2 suggests their potential involvement in AD pathogenesis. Focal adhesions are a type of cell–extracellular matrix adhesion structure that plays a key role in biological processes such as cell movement, proliferation, differentiation, and signal transduction (Stupack and Cheresh, 2002; Mitra et al., 2005). Additionally, focal adhesions can regulate Aβ signal transduction and cell death in cases of AD (Caltagarone et al., 2007). The synaptic vesicle cycle refers to the process by which presynaptic cells store, release, and reuptake neurotransmitters in synaptic vesicles. This process is essential to ensure the release of neurotransmitters and to maintain synaptic plasticity (Sudhof, 1995); furthermore, it has been a popular area of research related to neurodegenerative diseases (Wang et al., 2017). The tight junction is a pathway enriched in Cluster 1 that critically influences the maintenance of the integrity of the blood–brain barrier (Liu et al., 2012). AD can reportedly affect the blood–brain barrier function in *in vitro* experiments by altering the expression and localization of tight junction proteins (Desai et al., 2007). Impaired blood–brain barrier function in patients with AD may affect the clearance of Aβ, thereby accelerating AD progression (Algotsson and Winblad, 2007). The Wnt signaling pathway is a unique pathway enriched in cluster 3 that plays a crucial role in regulating adult brain structure and function. Downregulation of Aβ-induced Wnt signaling is associated with disease progression in AD, and activation of Wnt signaling can mitigate Aβ neurotoxicity and protect neurons. Therefore, the Wnt signaling pathway may be an important target for designing therapeutic strategies for AD in the future (Inestrosa and Varela-Nallar, 2014; Wan et al., 2014; Tapia-Rojas and Inestrosa, 2018).

Weighted gene co-expression network analysis helped in the identification of hub genes associated with each subtype and inputted the differentially expressed hub genes into the cMap database to screen for small-molecule drugs. LY-278584 may be a potential small molecule drug for cluster 1, Cetraxate for cluster 2, and Embelin for cluster 3. LY-278584 is a type of 5-HT_3_ receptor antagonist, and the 5-HT_3_ receptor is a neurotransmitter-gated ion channel located on the cell membrane that is highly expressed in the entorhinal cortex, hippocampus CA1 area, amygdala, substantia nigra, and brainstem. Antagonists of the 5-HT_3_ receptor are commonly used to treat nausea and vomiting and have also been found to have therapeutic effects on psychiatric disorders such as epilepsy, schizophrenia, and anxiety (Thompson and Lummis, 2007; Zhao et al., 2018). Importantly, 5-HT_3_ receptor antagonists may also delay memory impairment and enhance cognitive function, making them potential drugs for improving memory impairment in patients with AD (Fakhfouri et al., 2019). Cetraxate is a mucosal protective agent that can inhibit the activity of *H. pylori* and is commonly used as an anti-ulcer and anti-*H. pylori* drug (Kamada et al., 2000; Wu et al., 2004). Although there are currently no reports of cetraxate being used to treat AD, *H. pylori* may play a negative role in the development of AD, as it may enter the brain by disrupting the blood–brain barrier through circulating mononuclear cells and cause neurodegeneration (Doulberis et al., 2018). Further research is needed to determine whether cetraxate can be used to treat AD. Embelin is a natural product that can inhibit the aggregation of amyloid proteins and reduce inflammation. In addition, it can cross the blood–brain barrier and has antioxidant properties, which can prevent neuronal oxidative damage by reducing lipid peroxidation, thereby highlighting its potential as a therapeutic drug for AD (Nuthakki et al., 2019; Arora et al., 2023). Furthermore, embelin has been reported to be a potential drug for treating COVID-19 (Singh et al., 2021).

## 5. Conclusion

This study provides new evidence linking changes in MRGs expression levels to AD development and progression. The outcomes of the present study demonstrated that the overall expression of MRGs is downregulated in patients with AD, and six genes—i.e., *FUNDC1*, *MAP1LC3A*, *CSNK2A1*, *VDAC1*, *CSNK2B*, and *ATG5*—were identified that can be used to construct an AD prediction model with high predictive ability across different datasets. Furthermore, AD was classified into three subtypes based on MRGs expression profiles and the differences in gene expression, clinical features, immune infiltration, and pathway enrichment were analyzed among these subtypes. Despite our study is subject to limitations including relatively small sample sizes and a lack of experimental validation, the high heterogeneity of AD may lead to mitochondrial autophagy not occurring in all patients. Nonetheless, the results of the current study provide novel insights into the mechanisms underlying AD and emphasize the potential utility of MRGs in the diagnosis of and personalized treatment for AD.

## Data availability statement

The original contributions presented in the study are included in the article/Supplementary material; further inquiries can be directed to the corresponding authors.

## Author contributions

XG and CL conceived and designed the study. WM, YS, and PZ were responsible for the collection and assembly of data, data analysis, and interpretation. WM and YS were involved in the writing of the manuscript. GW, XC, and XG provided help in revising the manuscript. All authors contributed to the article and approved the submitted version.

## Funding

This work was supported by the National Natural Science Foundation of China (81772829 and 81830052), the Special Program for Collaborative Innovation, the Construction project of Shanghai Key Laboratory of Molecular Imaging (18DZ2260400) and “Top-100 Talent Cultivation Plan” of Shanghai University of Medicine and Health Sciences, and Funding Scheme for Training Young Teachers in Shanghai Colleges.

## Conflict of interest

The authors declare that the research was conducted in the absence of any commercial or financial relationships that could be construed as a potential conflict of interest.

## Publisher’s note

All claims expressed in this article are solely those of the authors and do not necessarily represent those of their affiliated organizations, or those of the publisher, the editors and the reviewers. Any product that may be evaluated in this article, or claim that may be made by its manufacturer, is not guaranteed or endorsed by the publisher.

## Footnotes

^1^https://www.ncbi.nlm.nih.gov/geo/

^2^https://clue.io/query
