# Supplementary material for: Identification of mitochondrial-related genes as potential biomarkers for the subtyping and prediction of Alzheimer’s disease
Source: Front Mol Neurosci. 2023 Jul 4;16:1205541. doi: 10.3389/fnmol.2023.1205541 (PMC10352499; doi:10.3389/fnmol.2023.1205541)
Supplement: Supplementary file 5 [file Presentation_1.pdf]

frontiers

Supplementary Material

Identification of mitochondrial-related genes as potential biomarkers for the subtyping and prediction of Alzheimer's disease

Wenhao Ma[1,2,3†], Yuelin Su[4†], Peng Zhang[2], Guoqing Wan[1,2], Xiaoqin Cheng[5], Changlian Lu[1,2*], Xuefeng Gu[1,2*]

[1] School of Pharmacy, Shanghai University of Medicine and Health Sciences, Shanghai, China

[2] Shanghai Key Laboratory of Molecular Imaging, Shanghai University of Medicine and Health Sciences, Shanghai, China

[3]School of Health Science and Engineering, University of Shanghai for Science and Technology, Shanghai, China

[4]Department of Ultrasound Medicine, Huashan Hospital Affiliated to Fudan University, Shanghai, China

[5]Department of Neurology, Zhongshan Hospital, Fudan University, Shanghai, China

†These authors share first authorship.

*** Correspondence:**
Xuefeng Gu
guxf@sumhs.edu.cn

Changlian Lu
lvcl@sumhs.edu.cn

1 Supplementary Figures

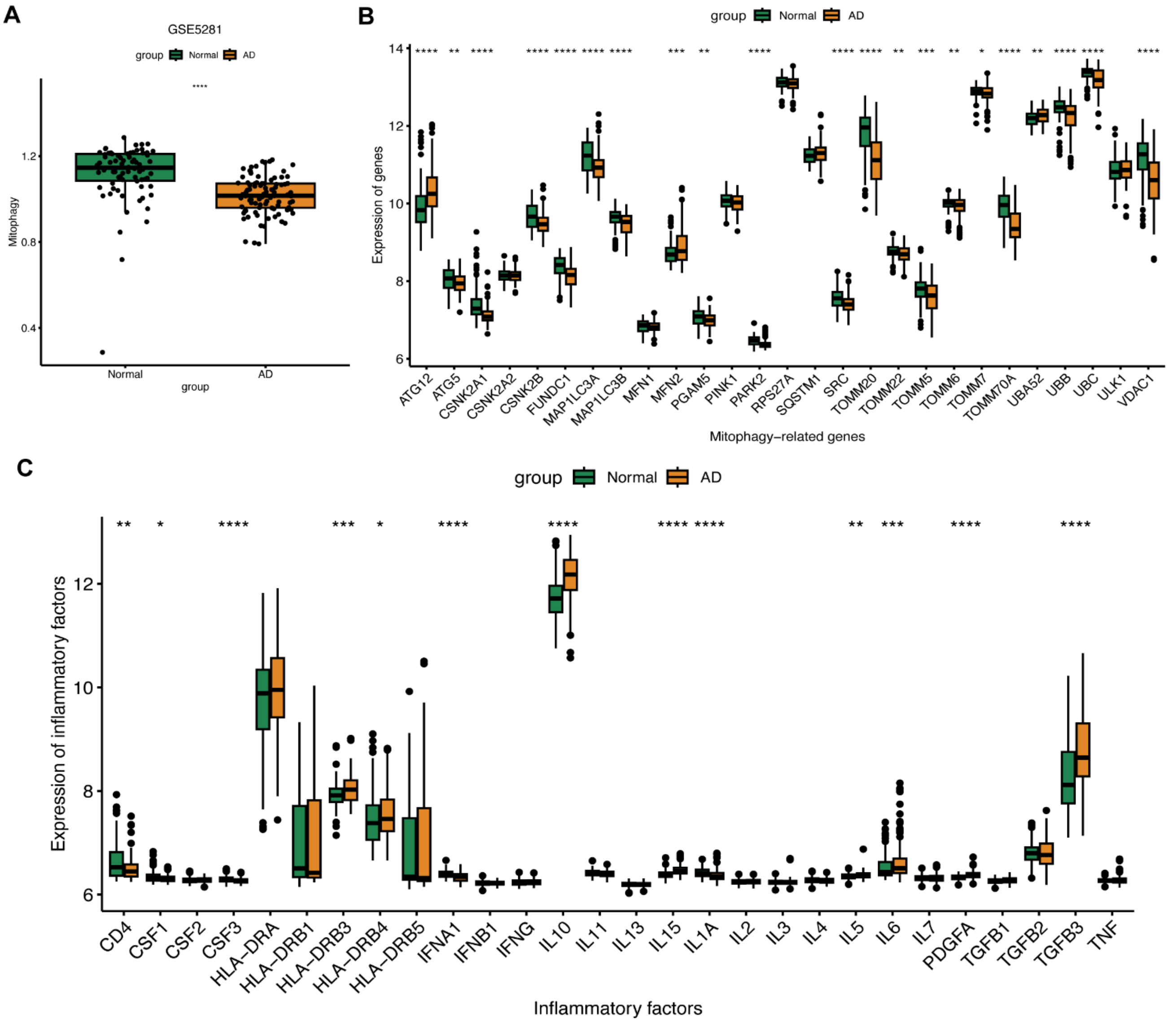

Supplementary Figure 1. Expression of MRGs and inflammatory factors in samples from healthy individuals and patients with AD. **(A)** Validation of overall expression changes of MRGs between samples from patients with AD and healthy individuals in a validation set (GSE5281). **(B)** Differential expression of specific MRGs between samples from patients with AD and healthy individuals (GSE109887&GSE132903). **(C)** Differential expression of inflammatory factors between samples from patients with AD and healthy individuals (GSE109887&GSE132903). P-values were estimated by Student's t-test. **P < 0.05, **P < 0.01, ***P < 0.001, ****P < 0.0001.*

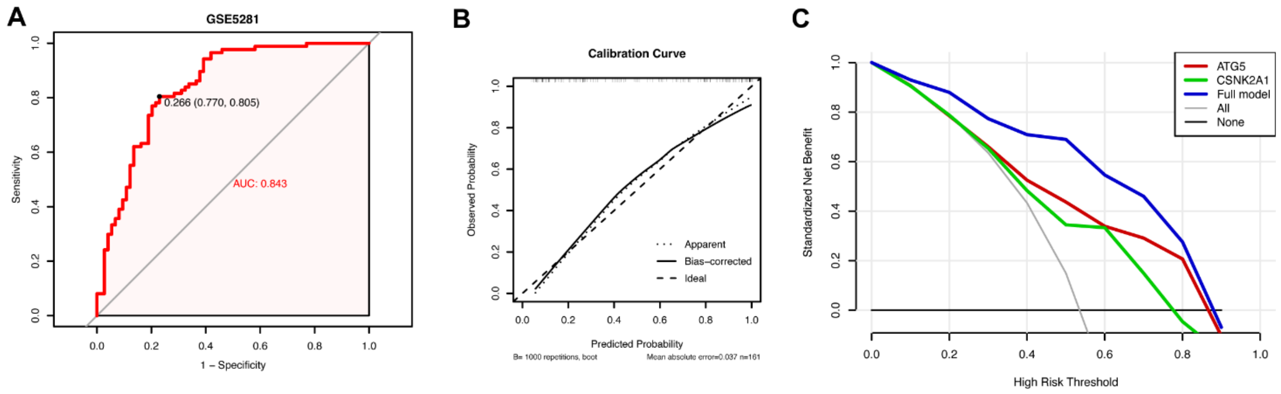

Supplementary Figure 2. Validation of the prediction model by the validation set GSE5281. **(A)** Receiver operating characteristic curve evaluating the diagnostic performance of feature genes. **(B)** Calibration curve illustrating the calibration performance of a predictive model. **(C)** DCA estimates the clinical benefit of the nomogram.

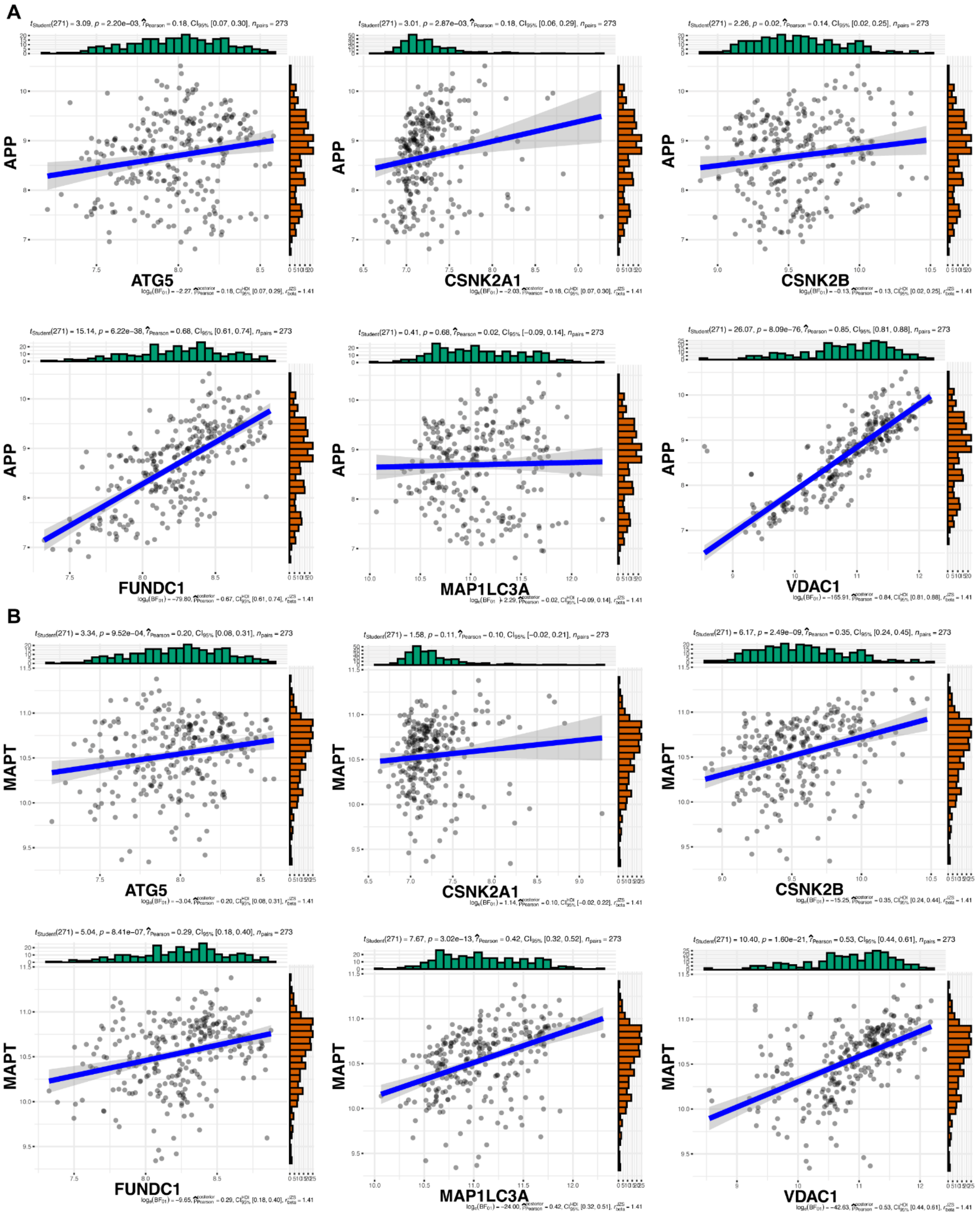

Supplementary Figure 3. Correlation of feature genes and amyloid precursor protein (APP) and tau protein (MAPT). **(A)** The correlation between the expression levels of six feature genes (*ATG5*,

CSNK2A1, *CSNK2B*, *FUNDC1*, *MAP1LC3A*, and *VDAC1*) and the expression levels of *APP*. **(B)** The correlation between the expression levels of six feature genes (*ATG5*, *CSNK2A1*, *CSNK2B*, *FUNDC1*, *MAP1LC3A*, and *VDAC1*) and the expression levels of *MAPT*.

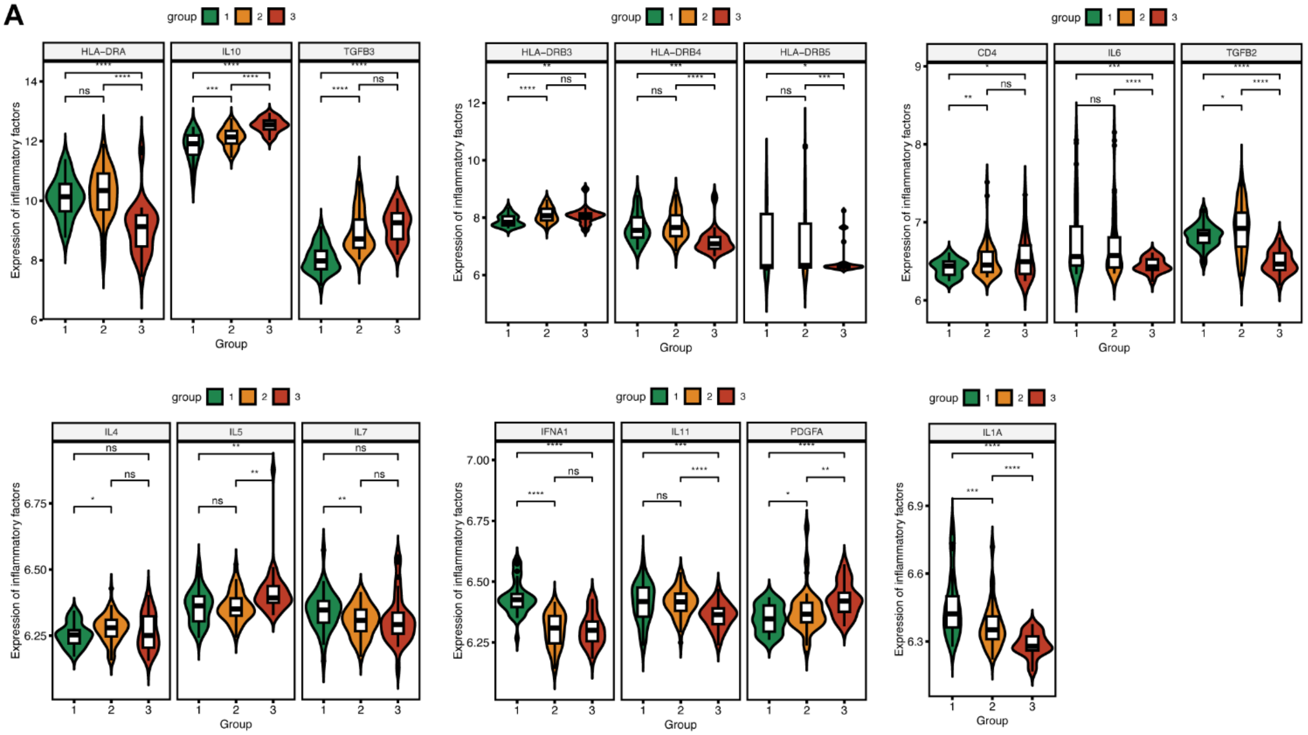

Supplementary Figure 4. (A) Differential expression of inflammatory factors in the three subtypes. P-values were estimated by Student's t-test.*$P < 0.05$, **$P < 0.01$, *** $P < 0.001$, **** $P < 0.0001$; ns, no significance.

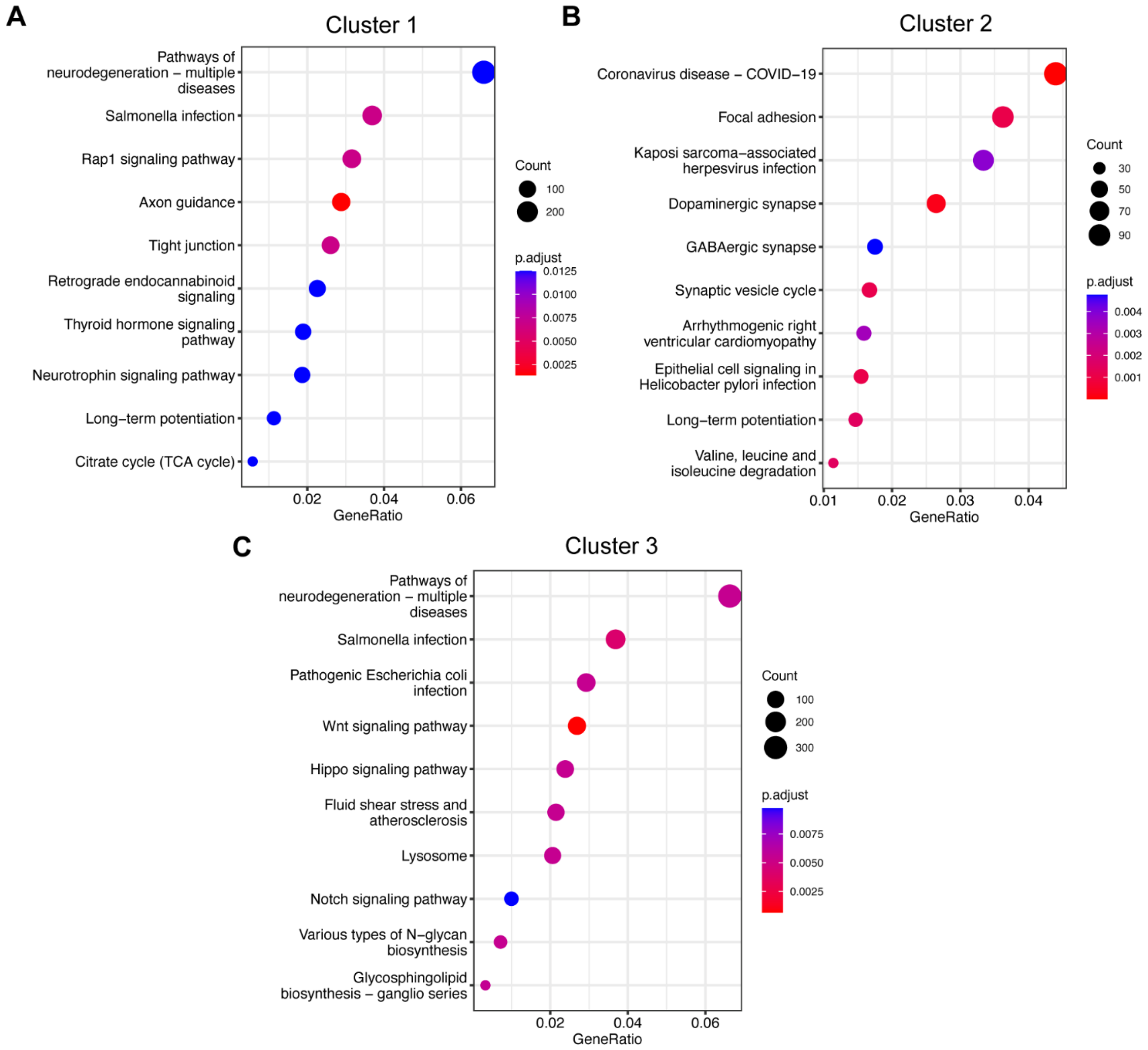

Supplementary Figure 5. Bubble chart of the top ten KEGG enriched pathways in each of the three subtypes of Alzheimer's disease. The size of the bubbles indicates the number of differentially expressed genes enriched in each signaling pathway, while the color reflects the corresponding P values. (A)Top ten KEGG enriched pathways in Cluster 1. (B) Top ten KEGG enriched pathways in Cluster 2. (C) Top ten KEGG enriched pathways in Cluster 3.
